# Origins of the Endogenous and Infectious Laboratory Mouse Gammaretroviruses

**DOI:** 10.3390/v7010001

**Published:** 2014-12-26

**Authors:** Christine A. Kozak

**Affiliations:** Laboratory of Molecular Microbiology, National Institute of Allergy and Infectious Diseases, Bethesda, MD 20892, USA; E-Mail: ckozak@niaid.nih.gov; Tel.: +1-301-496-0972; Fax: +1-301-480-6477

**Keywords:** mouse endogenous retroviruses, mouse leukemia viruses, house mouse subspecies, ecotropic/xenotropic/polytropic gammaretroviruses, retrovirus restriction factors, recombinant mouse gammaretroviruses

## Abstract

The mouse gammaretroviruses associated with leukemogenesis are found in the classical inbred mouse strains and in house mouse subspecies as infectious exogenous viruses (XRVs) and as endogenous retroviruses (ERVs) inserted into their host genomes. There are three major mouse leukemia virus (MuLV) subgroups in laboratory mice: ecotropic, xenotropic, and polytropic. These MuLV subgroups differ in host range, pathogenicity, receptor usage and subspecies of origin. The MuLV ERVs are recent acquisitions in the mouse genome as demonstrated by the presence of many full-length nondefective MuLV ERVs that produce XRVs, the segregation of these MuLV subgroups into different house mouse subspecies, and by the positional polymorphism of these loci among inbred strains and individual wild mice. While some ecotropic and xenotropic ERVs can produce XRVs directly, others, especially the pathogenic polytropic ERVs, do so only after recombinations that can involve all three ERV subgroups. Here, I describe individual MuLV ERVs found in the laboratory mice, their origins and geographic distribution in wild mouse subspecies, their varying ability to produce infectious virus and the biological consequences of this expression.

## 1. Introduction

Mouse leukemia viruses (MuLVs) of three host range subgroups are found in the common inbred strains of laboratory mice (Table 1). The mouse-tropic or ecotropic MuLVs (E-MuLVs) were discovered in these common inbred strains more than 60 years ago when it was found that extracts from mouse hematopoietic neoplasias could induce leukemias or lymphomas in inoculated animals [1]. Xenotropic MuLVs (X-MuLVs) were later isolated by Levy and Pincus from the NZB mouse strain [2], and were termed “xenotropic” because they could infect cells of multiple species, such as human, rabbit and cat, but were unable to infect cells of the mice from which they were isolated [3,4,5]. The third MuLV host range group, the polytropic MuLVs (P-MuLVs), can be isolated from mouse lymphomas and leukemias, and are also termed mink cell focus-forming (MCF) MuLVs because they can be cytopathic in mink lung cells. P-MuLVs were initially determined to be infectious in mouse cells as well as cells of heterologous species [6,7]. While these early observations suggested that P-MuLVs have the broadest host range of the MuLV subgroups, more recent studies have shown that X-MuLVs can actually infect more mammalian species than P-MuLVs, and that X-MuLVs but not P-MuLVs are capable of infecting all wild mouse taxa [8] (Table 1).

**Table 1:** Host range variants of mouse leukemia viruses isolated from laboratory mice.

Type	No. of ERV Copies in C57BL	Ability of XRVs to Infect Mouse and Other Mammalian Cells						Receptor
		Mouse				Mink, Human	Bat, Dog	
		Laboratory strains	*M. m. domesticus*	*M. m. castaneus*	*M. m. musculus*			
Ecotropic	1	+	+	+	+	-	-	CAT-1
Polytropic	>30	+	+	-	-	+	-	XPR1
Xenotropic	>20	-	+	+	+	+	+	XPR1

Infectious exogenous viruses (XRVs) of all three MuLV subtypes can be reliably isolated from various common strains of laboratory mice, and these strains also carry germline copies as endogenous retroviruses (ERVs) of all three subtypes. In this review, I will describe the distribution of MuLV ERVs in laboratory strains and in their wild mouse progenitors, the wild mouse origins of the individual MuLV ERV insertions found in the sequenced C57BL mouse genome, the different abilities of the various ERVs to produce XRVs, and the biological consequences of this expression.

## 2. Mouse Gammaretrovirus Genome

**Figure 1:**
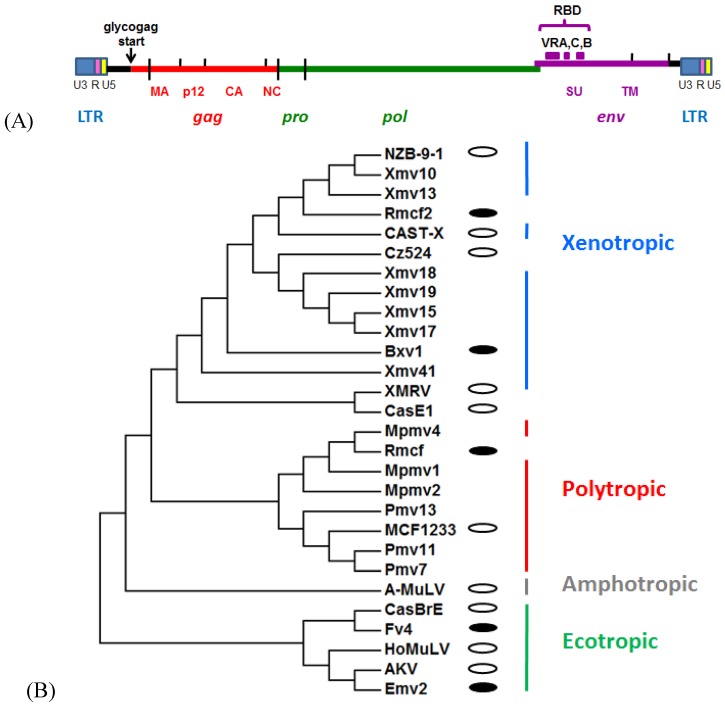
Endogenous and exogenous MuLVs. (**A**) Genomic structure of MuLV ERVs. (**B**) Phylogenetic tree of MuLV *env* receptor binding domains (RBDs) constructed using the neighbor-joining method [11] and inferred from 500 replicates using MEGA6 [12]. White ovals represent exogenous viruses; black ovals are active ERVs that produce viral proteins or infectious viruses; the rest are ERVs with unknown expression.

The MuLVs and their endogenous ERV counterparts, along with the alpharetroviruses, have the simplest of retrovirus genomes among the seven retroviral genera [9] (Figure 1A). These genomes encode the virus core proteins (*gag*), the enzymes necessary for replication (*pro, pol*) and the envelope glycoprotein (*env*). In ERVs, these protein-coding regions are flanked by long terminal repeat sequences (LTRs) that contain the regulatory elements needed for transcription. The gammaretroviruses do not encode additional accessory proteins as do the more complex retroviruses like HIV-1. MuLVs have only one zinc-finger in their *gag* nucleocapsid and they translate pol by reading through the in-frame *gag* termination codon. Gammaretroviruses are the only retroviruses that produce glyco-gag, a second, longer and glycosylated form of the Gag precursor polyprotein that is initiated at an alternate, upstream start site [10].

The MuLV host range subgroups are determined by sequence variation and receptor usage (Table 1, Figure 1B). The MuLV receptor-binding domain (RBD) of the Env glycoprotein is responsible for binding to specific host cell receptors [13,14,15]. The E-XRVs use the CAT-1 receptor for entry [16] and the X-XRVs and P-XRVs (together termed XP-XRVs) both use the XPR1 receptor [17,18,19] (Table 1). All gammaretroviruses use multipass transmembrane proteins as receptors, most of which function as small solute transporters; CAT-1 has been identified as the SLC7A1 amino acid transporter [20] while XPR1 has been implicated in signal transduction, as well as phosphate export [21,22]. Receptor choice is primarily determined by the first of the variable domains in the RBD, termed VRA, but sequences outside VRA also influence tropism [13,15,23,24]. MuLV host range is also affected by sequence polymorphisms in the host cell CAT-1 and XPR1 receptors and naturally occurring receptor variants account for different virus restriction patterns in mammalian species [8,25,26].

In addition to the prototypical E- and XP-MuLVs found in laboratory mice, sequence and host range MuLV variants have been identified in wild mouse species. These wild mouse isolates include some that rely for entry on the receptors CAT-1 (CasBrE, HoMuLV) or XPR1 (CasE#1, Cz524) [27,28,29,30], as well as viruses of amphotropic (A-MuLV) host range that are only found in wild mice of California, and the related 10A1 MuLV; A-MuLV and 10A1 use the PiT-1 and/or PiT-2 receptors [31,32,33,34].

Additional, but more distantly related mouse gammaretroviruses with ecotropic and nonecotropic host range have been identified in wild mice [35]. The viruses with ecotropic host range include HEMV, identified in *M. spicilegus* [36], and M813 from Asian *M. cervicolor* mice [37,38], both of which use the transporter SMIT1 as receptor rather than the E-MuLV receptor CAT-1 [39,40]. Another Asian mouse gammaretrovirus, McERV, shows sequence similarity to gibbon ape leukemia virus (GALV) which uses PiT-1 as receptor while the McERV receptor is plasmolipin (PLLP) [41]. Other mouse gammaretroviruses, such as MDEV and GLN-2, use novel but unidentified receptors [42,43].

This review will focus on the subgroups of MuLVs isolated as infectious viruses from the various laboratory mice, that is, the ecotropic and nonecotropic viruses that use either CAT-1 or XPR1 for entry.

## 3. Laboratory Strains and Wild Mouse Species that Carry MuLV ERVs

**Figure 2:**
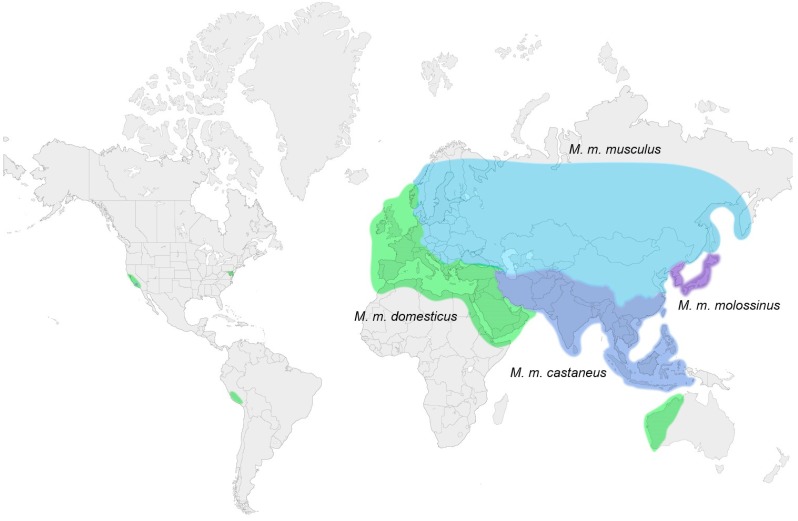
Geographic distribution of XP-MuLV infected house mouse subspecies. The three blue color blocks represent the ranges of the three subspecies carrying predominantly X-MuLVs; green represents P-MuLV infected *M. m. domesticus*. Wild-caught American house mice are largely *domesticus*.

The common strains of laboratory mice are genetic mosaics of the house mouse subspecies of wild mice. In the genus *Mus*, there are 40 species in four subgenera, and the most recent radiation in the *Mus* subgenus generated the three major lineages of commensal or house mice: *Mus musculus musculus, domesticus, castaneus* [44]. These mice are termed house mice because they are dependent on humans and live in human-built structures like houses, ships and warehouses [45]. House mice originated on the Indian subcontinent 0.5–1.0 mya [46] and then spread throughout Eurasia where the various house mouse subspecies have largely nonoverlapping geographical ranges [47]. These mice also accompanied humans to the Americas where *M. m. domesticus* of Western Europe predominates (Figure 2). Over the course of the last several hundred years, these house mouse subspecies were interbred by hobbyists who maintained colonies of fancy mice as pets and for show, and these fancy mice were eventually used to develop the common strains of laboratory mice [48,49].

Retroelements comprise 37% of the sequenced genome of the C57BL mouse, and 8-10% of this genome consists of ERVs [35,50] which originated as insertions generated by past retroviral infections. There are three classes of ERVs in the mouse genome. The Class III ERVs are the most ancient and most abundant and include the ERV-L elements that remain active in mice but not humans. The Class II ERVs are the second most abundant and include mouse mammary tumor viruses (MMTVs) and intracisternal particles (IAPs). The gammaretroviruses, including the MuLVs, are members of the smallest class, Class I, which represents only 0.7% of the mouse genome. E-MuLVs and XP-MuLVs are present as ERVs in laboratory mice, and these germline integrations were initially identified by Southern blot analysis of genomic DNA using *env-*derived probes that distinguish these 3 host range groups [51,52,53]. Many, but not all, of the common strains of laboratory mice carry a small number of E-MuLV ERVs but all strains carry dozens of germline copies of the XP-MuLVs [54] (Table 1). All 3 MuLV *env* subgroups are also found in various *Mus* taxa but are largely restricted to the house mouse subspecies.

### 3.1. Ecotropic MuLV ERVs

Three E-MuLV subtypes have been isolated from laboratory strains and wild mouse species (Table 1). The E-XRVs first isolated from laboratory mice are the AKV-type, named after the virus isolated from the high titer virus-producing strain AKR. There are more than 30 distinct germline copies of the AKV E-MuLV *env* genes in laboratory mouse strains, termed *Emvs*, many of which are shared by inbred strains with common ancestry [51]. While some strains carry no *Emvs*, like SJL and other NIH Swiss-derived strains, some strains, like C58/J, can carry six or more. AKV *Emv*-related ERVs have also been found in some house mouse subspecies [55]. These ERVs are carried by the Japanese house mouse *M. m. molossinus* which is a natural hybrid of *M. m. castaneus* and *M. m. musculus* [56], and have also been found in mice trapped in Korea and Northern China [57]. 

CasBrE MuLV is the prototype of the second E-MuLV subtype and was isolated from California wild mice. This virus has ecotropic host range, and uses the same CAT-1 cell surface receptor for entry, but its *env* RBD shows only about 68% identity to that of AKV [27]. The CasBrE *env* shows closer identity (89%) to that of *Fv4*, a virus restriction gene that encodes an MuLV *env* [58,59,60] (Figure 1B). CasBrE-related ERVs and the *Fv4* gene are found in *M. m. molossinus* and *M. m. castaneus* mice [57], and are also present in localized populations of California wild mice trapped in sites, such as Lake Casitas; these mice are likely derived from the interbreeding of Asian wild mice that accompanied Chinese laborers coming to America and *M.m. domesticus* brought by colonists from Western Europe [55,57,61].

The third E-MuLV subtype, HoMuLV, was isolated from the Eastern European mouse, *Mus spicilegus* (formerly *M. hortulanus*) [62], a species that is not in the house mouse complex. HoMuLV also uses CAT-1 and its *env* RBD is 68% and 66% identical to AKV and CasBrE, respectively [28] (Figure 1B). Unlike AKV and CasBrE, which are endogenous in *Mus*, HoMuLV is a horizontally transmitted virus that has not been identified as an ERV in any mouse [28].

### 3.2. Xenotropic/Polytropic MuLV ERVs

All inbred strains of laboratory mice carry XP-MuLV ERVs, and Southern blot analysis identified up to 57 XP-MuLV copies in the different classical inbred strains, although some, like SWR, have few X-MuLV ERVs [52,63,64]. There are three subtypes of XP-MuLV ERVs in laboratory mice: xenotropic ERVs termed *Xmvs*, and two closely related subtypes of P-MuLV ERVs distinguished as polytropic (*Pmvs*) and modified polytropic (*Mpmvs*) (Figure 1B) [53]. The chromosomal locations of multiple individual *Xmvs*, *Pmvs*, and *Mpmvs* were identified in several laboratory strains by conventional genetic methods [64]. While the XP-MuLVs tend to be stable genetic elements, they show insertional polymorphism among the inbred strains and substrains with the occasional acquisition of novel insertions observed during the inbreeding of recombinant inbred lines [63,64,65] and the occasional loss of full-length ERVs by homologous recombination leaving behind solo LTRs [66,67]. The proviral genomes of the individual *Xmvs* in the sequenced C57BL genome are more polymorphic than the P-MuLV ERVs and this diversity may have been derived from several separate infections, and may also reflect the greater age of this subgroup [68,69,70] (Figure 1B).

In *Mus* taxa, the XP-MuLV ERVs are more widely distributed and are present in greater copy number than E-MuLV ERVs. XP-MuLV ERVs are found in all house mice subspecies, but the different subtypes are largely segregated into different subspecies [55] (Figure 2). P-MuLV ERVs but not *Xmvs* are found in *M. m. domesticus* of Western Europe. P-MuLV ERVs are also found in *M. spretus*, but the few copies in this species were likely derived from introgression resulting from limited interbreeding with sympatric *M. m. domesticus* [71,72]. *Xmv*s predominate in *M. m. castaneus, M. m. molossinus* and *M. m. musculus* of Eastern Europe and Asia. Screens for subtype-specific sequences from the viral LTR and from *env* segments outside the RBD confirmed this pattern of XP-MuLV subtype segregation in house mouse subspecies, and also identified recombinant types not found in inbred strains [36,73]. Because the western European *M. m. domesticus* is found in the Americas, American mice therefore carry P-MuLV ERVs [55], although the Lake Casitas mice differ from other American house mice in that they also carry multiple copies of *Xmv*s likely acquired from their interbreeding with Asian mice [55].

Some of the individual XP-MuLV ERVs found in the sequenced genome of C57BL have been traced to wild mouse subspecies [66,74,75] (Table 2). The C57BL *Xmv* ERVs are all present in *M. m. molossinus* Japanese mice, and two of these *Xmvs*, *Xmv42 and Xmv8*, were also found in *M. m. castaneus*. A 13^th^ X-MuLV ERV, preXMRV-1, present in some inbred strains but not C57BL, was also traced to *M. m. molossinus* and *M. m. castaneus* [76]. This indicates that these particular *Xmvs* were introduced into laboratory strains from their Asian progenitors, and, in fact, Japanese mice were included in the fancy mouse colonies used to develop the classical inbred strains [77,78]. Although *Xmvs* clearly predate the origins of laboratory mice and the *M. m. molossinus* hybrids, that is not the case for the *Pmvs* and *Mpmvs* in the C57BL genome, none of which were found in any wild mouse, including *M. m. domesticus.* This is surprising since P-MuLV ERVs originated in this subspecies and the laboratory mouse genome is 95% *M. m. domesticus* [79]. Further examination of inbred strains having shared P-MuLV insertions showed that these sites are found in regions of shared haplotype and thus predate the development of the laboratory strains [66]. Thus, the C57BL *Mpmvs* and *Pmvs* show a high degree of insertional polymorphism but were acquired before the generation of the inbred strains. Thus, either these P-MuLV insertions arose in *M. m. domesticus* populations that were not sampled in this attempt to identify their wild mouse origins or these ERVs originated in the fancy mice that gave rise to the laboratory strains.

**Table 2:** Presence or absence of 43 individual C57BL XP-MuLV ERVs in house mouse subspecies.

Type	Number of C57BL ERVs	Number Present in House Mouse Subspecies *			
		*M. m. molossinus*	*M. m. castaneus*	*M. m. musculus*	*M. m. domesticus*
*Xmvs*	12	12	2	0	0
*Pmvs*	19	0	0	0	0
*Mpmvs*	12	0	0	0	0

* [66].

## 4. Origins of Infectious MuLVs

Infectious viruses of all three MuLV host range groups can be readily isolated from the various common inbred mouse strains. In addition to host range differences, individual MuLV isolates differ phenotypically from one another in reactivity with anti-MuLV antibodies, cross-interference patterns and receptor use, susceptibility to host restriction factors, cytopathicity and pathogenicity in mice. Some of the X-MuLV and E-MuLV isolates are products of specific nondefective ERVs, while others, including all P-XRVs, are generated by recombination involving ERVs of different subgroups.

### 4.1. Active E-MuLV ERVs (Emvs)

Most of the laboratory mouse *Emvs* are full-length, functional proviruses or have small defects. (Table 3) [51]. The high virus laboratory strains like AKR carry several such nondefective *Emv*s that constitutively produce infectious virus from birth [80]. In these viremic strains, novel *Emv* proviruses can be acquired over time [81,82,83,84]; this phenomenon has been attributed to oocyte infection from viremic mothers [85].

The *Emvs* carried by many other mouse strains are inefficiently expressed, although this expression can be enhanced or induced by halogenated pyrimidines [86,87]. Mouse strains carrying these *Emv*s can produce infectious virus late in life (Table 3) [88,89,90]. Some of these poorly expressed *Emvs,* like *Emv1* and *Emv2,* encode viruses termed N-tropic that are restricted by the host *Fv1^b^* allele, and also carry minor but fatal defects which interfere with replication (Table 3). These small defects can be corrected by mutation or by recombinations that can result when different viral genomes are copackaged [91,92,93]. Similarly, B-tropic E-XRVs, that are restricted by *Fv1^n^* but not *Fv1^b^*, are produced in aging *Fv1^b^* BALB/c and C57BL mice; these B-tropic viruses are derived from the endogenous N-tropic *Emvs* carried by these mice but have acquired escape mutations in the *Fv1* target site in the virus capsid gene [94]. Additionally, mice carrying multiple *Emvs* having different defects can produce replication-competent virus efficiently as was shown in hybrid mice carrying both *Emv2* and *Emv1* [95], and in HRS mice that carry *Emv1* and *Emv3* [96].

### 4.2. Active X-MuLV ERVs (Xmvs)

Among the laboratory mice, two strains, NZB and F/St, produce high levels of X-XRVs throughout their lives [2,97,98] (Table 3). This expression results from constitutively expressed *Xmvs* as this virus cannot spread due to the mutated XPR1 receptor found in most common inbred strains. Other laboratory strains rarely produce X-XRVs, but cultured cells from many common strains can produce X-XRVs following chemical induction [99]. Stimulation of spleen cells by bacterial lipopolysacccharide or in a graft versus host reaction also activates expression of *Xmvs* [100,101].

**Table 3:** MuLV ERVs that produce infectious virus.

Type	ERV *	Expression Level	Mouse Strains	Chromosome	Defect	Reference
Ecotropic	*Emv1*	Low	BALB/c,CBA,C3H	5	Env: furin cleavage site	[102,103,104]
	*Emv2*	Low	C57BL	8	Pol mutation	[92,105]
	*Emv3*	Low	DBA	9	Gag: myristylation site	[106]
	*Emv10*	Low	SJL	?	None	[107]
	*Emv11*	High	AKR	7	None	[108]
	*Emv12*	High	AKR	16	None	[109]
	*Emv13*	Low	AKR	2	Env: C-terminus	[110]
	*Emv14*	High	AKR	11	None	[111]
	*Emv26*	High	C58	8	None	[105]
	*Emv30*	Low	NOD	11	None	[112]
Xenotropic	*Bxv1*	Low High	BALB,C57BL,AKR F/St	1	None	[75,113]
	*Mxv1*	Low	MA/My	?	?	[74]
	*Nzv1*	Low	NZB	?	?	[114]
	*Nzv2*	High	NZB	?	None	[114]

* Specific *Emvs* were identified by Southern blot analysis [51].

Laboratory mice carry at least four active *Xmvs* capable of producing virus (Table 3). The *Bxv1 Xmv*, also termed *Xmv43*, is carried by many of the common strains of inbred mice [75] and has been identified in the sequenced C57BL genome [75]. *In vivo* expression of *Bxv1* is low except in F/St mice, where its high expression is linked to the major histocompatibility locus [115]. The high virus NZB mouse carries two active *Xmvs* [74,114,116]. *Nzv2* is constitutively active*,* while *Nzv1* is poorly expressed [114]. MA/My carries the fourth identified active *Xmv* along with *Bxv1* [74].

Infectious XP-MuLVs that use the XPR1 receptor have been isolated from lymphoid tissues or cultured cells of mice from Eurasia and California, although these viruses have been incompletely characterized [29,30,74,117,118,119]. Using classical Mendelian crosses, *M. m. molossinus* was shown to carry several ERVs capable of producing X-XRVs [74], one of which is the active laboratory mouse *Xmv*, *Bxv1* [75]*.*

Viruses with xenotropic host range, like CAST-X, have been isolated from *M. m. molossinus* and *M. m. castaneus* and these viruses largely resemble their laboratory mouse counterparts [119,120] (Figure 1B). Other XP-XRVs isolated from wild mice are not classifiable as X- or P-MuLVs. One such virus, CasE#1, was isolated from a wild-trapped California mouse [29]. Like P-XRVs it can produce MCF-type foci and interferes with P-XRVs, but, like X-XRVs, CasE#1 fails to infect laboratory mouse cells and its receptor requirements distinguish it from prototypical X- and P-XRVs [29,30,119]. Cz524 MuLV was isolated from the wild-derived *M. m. musculus* strain CZECHII/EiJ, and it differs from both P- and X-XRVs in host range [30]. The *env* genes of these two wild mouse isolates are not identical to any infectious or endogenous laboratory mouse XP-MuLVs, but are clearly XP-MuLV-related [30,119] (Figure 1B). It is not known if ERV counterparts of these wild mouse viruses exist in the genomes of the house mouse subspecies.

### 4.3. Recombinant P-MuLVs Generated during Leukemogenesis

Although many *Pmvs* and *Mpmvs* have coding regions with open reading frames [68], none have been shown to be capable of producing infectious virus. The reason for this failure has not been determined, but may be due to LTR defects or to unidentified coding region replacement mutations that inhibit replication. All P-MuLV ERV LTRs contain a negative regulatory element [121] and a 190 base pair (bp) LTR insertion [122] that disrupts the U3 enhancer region. While there is some evidence that these LTRs have some transcriptional activity [123], no infectious viruses carry this 190 bp insertion. Another distinction between the P-MuLV ERVs and XRVs is the fact that the P-MuLV ERVs, along with some *Xmvs,* cannot produce glyco-gag, which is encoded by all infectious MuLVs with the single exception of XMRV [124].

Despite the apparent inability of P-MuLV ERVs to produce infectious virions, these ERVs have clearly undergone amplification in *M. m. domesticus*, although the responsible mechanism has not been determined. In the mouse strains viremic with E-XRVs, P-MuLVs are transmitted as pseudotypes in which the transcribed products of P-MuLV ERVs are packaged into E-MuLV virions [125,126,127]. P-MuLVs can also bypass their cognate receptor and use the E-MuLV CAT-1 receptor in the presence of the soluble E-MuLV RBD [128]. These transmission mechanisms, however, require the presence of E-MuLV virions or proteins, and there is no evidence of present or past infection by E-MuLVs in populations of *M. m. domesticus.*

The infectious MCF-type P-XRVs are generated in laboratory mice carrying replicating E-XRVs, and this process is linked to virus-induced lymphoma. The disease process, defined largely in AKR strain mice, also occurs in other mouse strains carrying multiple *Emv*s, like HRS and C58. Leukemogenesis begins with activation of germline *Emvs* or acquisition of infectious virus by horizontal transmission. As these E-XRVs establish a chronic infection, they recombine with endogenous XP-MuLVs to produce MCF-type viruses with P-MuLV host range and increased virulence [129,130]. These recombinant viruses can be identified as early as 3 weeks after birth [131] and most AKR mice die of virus-induced disease by six to nine months of age. P-MuLVs induce disease by insertional mutagenesis in which novel somatic viral integrations either activate genes like *Myc* that are involved in growth regulation or inactivate tumor suppressor genes like *Trp53* [132,133]. There is also evidence that the P-MuLV *env* substitutions influence target cell specificity and disease type [129], act as mitogens to induce T-cell proliferation in preleukemic tissues [134], or interfere with the immune response [135,136,137]. Also, the failure of P-MuLVs to establish superinfection interference results in a large amount of unintegrated MCF P-MuLV DNA and newly acquired proviruses in tumors [138,139] that has been linked to cytopathic killing [140] through endoplasmic reticulum stress-induced apoptosis [141].

The importance of MCF MuLVs in neoplastic disease is supported by the appearance of these viruses in pre-leukemic tissues, the presence of novel clonal integrations in tumors [138], and the acceleration of disease after their inoculation into neonatal AKR mice [142,143]. This association with disease is further supported by observations that disease is suppressed in mice carrying the *Rmcf* resistance gene that inhibits replication of P-XRVs [144] and is also blocked in mice inoculated with genetically altered viruses that cannot participate in MCF production [145].

The demonstration that P-XRVs are recombinants was based on peptide mapping, oligonucleotide fingerprinting, restriction mapping and partial sequencing [146,147,148,149,150]. All MCF recombinants have P-MuLV *env* sequences and many also have X-MuLV related LTRs contributed by the active *Bxv1 Xmv* [130,151,152]. The recombinant *env* segments in MCF MuLVs vary in sequence because different P-MuLV ERVs contribute *env* segments [148]. The acquired *env* sequence also varies in size [149], and sequencing shows that the recombinational breakpoints in the MCF *env* are clustered in three segments, two of which are in the 3’ half of SU*env* and one of which is in the 5’ end of TM*env* [71,153,154].

Infectious P-XRVs are generated *de novo* in each E-XRV infected mouse, and individual isolates vary in pathogenicity [142]. The leukemogenic potential of these P-XRVs is assessed by their ability to accelerate the onset of thymomas after inoculation into newborn AKR mice [142,143]. The virus isolates that have been judged to be lymphomagenic were isolated from tumor tissue of high leukemic mice, whereas P-XRVs from strains with a low incidence of disease are generally not lymphomagenic. Comparisons of pathogenic and nonpathogenic P-XRVs, and comparisons of P-XRVs from leukemic or preleukemic mice, found consistent sequence differences: the viruses from diseased mice tend to have *Xmv*-like LTRs and smaller P-MuLV ERV substitutions in *env* [130,155,156].

The wild mice that naturally carry P-MuLV ERVs but not E-MuLVs (*M. m. domesticus*) do not produce infectious P-MuLVs. These mice have a low incidence of leukemias and lymphomas, but studies on another species, *M. spretus,* which carry a few P-MuLV ERVs but no *Emvs* or *Xmvs*, showed these mice can, like laboratory mice, develop lymphomas after inoculation with infectious E-XRVs; disease is accompanied by the generation of replication competent recombinant P-XRVs [71].

### 4.4. XRVs in Xenografts and Immunosuppressed Mice

Some mice carrying specific immune deficiencies are characterized by the early activation of MuLV ERVs and by retrovirus-induced disease. In one study, the absence of the toll-like receptors, TLR3, -7 and -9, was associated with early detection of MuLV proteins and particles, and with the development of pre-T cell acute lymphoblastic leukemia induced through insertional mutagenesis [157]. A second study showed that *Rag1^-/-^* mice, which lack functional B and T cells, carry infectious E-XRVs that are vertically transmitted to their progeny and are associated with retrovirus-induced lymphomas [158]. In both studies, disease was linked to E-XRVs produced by *Emv2* and to P-XRVs derived by recombination involving *Emv2,* *Bxv1* and other nonecotropic ERVs.

Replication competent recombinant viruses have also been isolated from human xenografts passed in immunodeficient mice. The most notorious example is XMRV, a gammaretrovirus closely related to X-MuLVs, that was identified in the search for a viral pathogen associated with human prostate cancer [159]. XMRV is now recognized as a recombinant virus derived from two MuLV ERVs, the *Xmv*-like PreXMRV-1 and the P-MuLV-like PreXMRV-2. This recombinant was acquired by a xenografted human prostate tumor passaged in *nude* mice [160]. Some other xenografted human tumors have been found to carry other MuLVs including *Bxv1*-like X-MuLVs (for example [161]), which is not surprising as *Bxv1* is subject to induction by immunostimulation.

Induced expression of MuLV ERVs and the subsequent generation of recombinant viruses can have unanticipated phenotypic consequences in xenograft experiments designed to model human diseases. Mice homozygous for *Prkdc^scid^* and *Il2rg^null^* xenografted with human primary myelofibrosis showed a high frequency of acute myeloid leukemia that was of mouse origin [162]. The somatically acquired MuLV insertions in the mouse tumors were clonal, and integration was observed in *Evi1*, a common integration site for virus-induced myeloid leukemia [162,163]. The E-XRV recovered from tumors was derived from the E-MuLV ERV *Emv30*, and the tumors also contained some recombinants with P-MuLV *env* sequences. These infectious MuLVs were detected in diseased xenografted mice but also in tumor-free controls without xenografts indicating that active MuLV infection was not sufficient for disease induction. That pathogenicity is restricted to transplanted mice suggests that paracrine stimulation of mouse myeloid cells produced a proliferating target cell population that was then transformed by MuLV infection. Thus, xenografting may produce unexpected pathologies resulting from ERV activation.

### 4.5. The Generation of Acute Transforming Viruses

In addition to replication competent recombinant viruses that cause disease after a long latency period, recombination can also generate transforming retroviruses that cause disease after a short latency period [132]. Diseases induced by these viruses include sarcomas, erythroleukemia, and lymphomas. These viruses are pathogenic because they have transduced host cell proto-oncogenes. These acquired sequences almost always displace viral genes, and the resulting recombinants are not replication competent but require a helper virus for transmission. The tumors induced by these viruses are polyclonal and result from disruption of growth factor pathways by expression of the transduced gene. Discovery of these viruses has led to the identification of many cellular genes involved in oncogenesis. Examples of ERV-derived acute transforming viruses include Abelson MuLV which carries the *abl* oncogene and FBJ mouse sarcoma virus which carries *fos* [132]. While some of these recombinant viruses were derived from naturally occurring active *Emvs*, others were produced after inoculating mice with laboratory strains of XRVs like Moloney or Friend MuLVs. In one unusual case, the oncogenic activity of the Friend MuLV-derived spleen focus forming virus, SFFV, was mapped to its *env* gene [164,165] that induces acute erythroblastosis by stimulating the erythropoietin receptor and by activating the signal transduction pathway linked to the protein tyrosine kinase sf-Stk [166].

## 5. Co-Opted ERVs that Function as Antiretroviral Restriction Factors

After endogenization, ERVs acquire mutations that compromise their functionality, and progressive mutational degeneration eventually renders them unrecognizable as ERVs. In some exceptional cases the ERV regulatory or protein coding sequences can be co-opted by the host for cellular functions and can then be preserved by purifying selection. Some of these domesticated ERVs serve as antiviral restriction factors that interfere with exogenous virus infection. In fact, the first antiviral host restriction factor to be described, *Fv1,* is a co-opted ERV sequence related to the *gag* gene of the MuERV-L family [167,168,169]. *Fv1* is found only in *Mus* species, and targets the virus capsid of the mouse-tropic MuLVs to inhibit virus replication [170,171,172], and it can also restrict some non-MuLV retroviruses [173].

Specific MuLV ERVs have also been co-opted as host restriction genes (Table 4). Most of these retrovirus-related resistance genes encode MuLV Env glycoproteins that are thought to restrict the entry of exogenous virus. These genes include *Fv4*, which blocks E-XRVs [58], and the genes *Rmcf* and *Rmcf2* which both restrict P-XRVs but are derived from different XP-MuLV ERV subtypes [65,174] (Figure 1B). *Fv4* and *Rmcf* have also been shown to inhibit MuLV-induced disease [175,176,177]. The ERVs mapped to these resistance genes are defective for virus production but have intact *env* genes [58,65,174], and there is evidence for other such genes in Asian house mice [5]. *Fv4*, *Rcmf*, and *Rcmf2* may function through receptor interference, but *Fv4* additionally has a defect in its *env* fusion peptide, so virus-infected cells carrying *Fv4* produce virions that incorporate this Env and have reduced infectivity [178]. Comparable co-opted *env* genes with antiviral activity are also found in chickens, sheep and cats indicating that this is a common and effective antiviral strategy in natural populations exposed to infectious retroviruses [179,180,181].

**Table 4:** MuLV ERVs associated with restriction of exogenous MuLVs.

Restriction Gene	Progenitor MuLV	ERV Structure	Restricted virus	Distribution	
				Inbred Strains	*M. musculus* subspecies
*Fv4*	CasBrE E-MuLV	*env* and 3’LTR	E-MuLV	G	*castaneus, molossinus*, Lake Casitas mice
*Rmcf*	XP-MuLV	Deletion spanning *gag,pol*	P-MuLV	DBA/2, CBA	-
*Rmcf2*	XP-MuLV	Termination codon in *pol*	P-MuLV	-	*castaneus*
*Apobec3*	X-MuLV	Solo LTR	E-MuLV	C57BL,NZB,RIIIS	*musculus*

While retroviral insertions add protein-coding sequences to the host genome like the *Fv1* capsid and *Fv4* *env*, ERVs can also introduce regulatory elements that affect host gene expression. It has been estimated that >10% of new mouse mutations are due to ERV insertions [182], and enhanced expression of one host restriction gene has been linked to one such MuLV insertion. Mouse *Apobec3* (mA3) encodes a cytidine deaminase that is packaged in virions and restricts virus in newly infected cells by introducing G>A mutations in the reverse transcribed viral DNA, or through an unknown mechanism that interferes with reverse transcription [183,184]. There are two alleles of mA3 in laboratory mice, and the C57BL mA3 is more effectively antiviral than the BALB/c allele due to differences in expression level, protein sequence and splicing pattern [185]. C57BL and the other inbred strains and wild mouse species with increased mA3 expression have an X-MuLV LTR inserted into an mA3 intron [186]. LTRs can enhance activity of nearby cellular promoters, and this provides an explanation for the elevated mA3 expression that is unique to the various LTR+ inbred strains and wild mice.

## 6. Horizontal and Trans-Species Transmission

MuLVs are blood-borne pathogens that have a broad host range (Table 1), and their receptors have a ubiquitious tissue expression pattern. The horizontal transfer of infectious MuLVs between individuals has been documented in wild mouse populations and in laboratory mice [187,188,189]. In mice, such transfers have been found to occur through fighting, mating, suckling and by transplacental infection. Some of the MuLVs found in wild mouse populations, like HoMuLV E-MuLV and A-MuLV, are carried only as infectious agents that have not endogenized [28,190].

MuLV-infected house mouse species have a global distribution [47], and mice are recognized pathogen reservoirs and disease vectors [191]. It is therefore not surprising that rodent-related gammaretroviral ERVs have been found in the genomes of amphibians, reptiles, birds and mammals, and that trans-species transmission of rodent retroviruses has been a common occurrence in mammalian evolution [192,193]. Successful interspecies transmissions can produce disease in new hosts that are unprepared to resist unfamiliar and potentially pathogenic agents [194]. Although there is no evidence of the transmission of the prototypical laboratory mouse MuLV gammaretroviruses to other species, the pathogenic GALV and KoRV (koala retrovirus) retroviruses found in natural and zoo populations are related to Asian mouse gammaretroviruses [195]. In some cases, newly introduced pathogenic viruses can be inhibited by host restriction genes. Thus, studies on XMRV infected cultured human peripheral blood mononuclear cells and pigtailed macaques indicate that virus replication is restricted mainly by APOBEC-mediated hypermutation [196,197]. It has also been observed that species exposed to exogenous MuLVs can acquire protective mutations that inhibit infection. This is the case for mouse taxa carrying infectious MuLVs; the Asian *M. musculus* subspecies that harbor X- and E-XRVs have acquired *env* genes that restrict MuLV entry, XPR1 and CAT-1 receptor mutations responsible for restrictive phenotypes, and more restrictive mA3 alleles [198]. Similar mutational mechanisms may operate to restrict trans-species transmissions. It has thus been shown that fowl and raptor avian species found in geographic areas populated by X-MLV infected mice are more resistant to XP-XRVs due to XPR1 mutations at the same sites mutated in the disabled laboratory mouse XPR1 receptor [199].

## 7. Conclusions

MuLVs are simple retroviruses in the gammaretrovirus genus that have long received a lot of attention because of their links to neoplastic, immunological and neurological diseases, and because of their demonstrated potential for trans-species transmission. Studies on the mechanisms underlying retrovirus-host interactions have greatly benefited from this early focus on MuLVs because of the availability of inbred strains having different complements of MuLV ERVs and with different disease profiles, as well as the existence of globally distributed wild mouse populations carrying different MuLV subgroups and with different host restriction factors [197]. Analysis of these strains and species have found biologically distinct infectious and endogenous viruses in these mice, identified processes and multiple host factors involved in virus transmission and virus-induced disease, and described the acquisition and amplification of ERVs, and the appearance and evolution of host restriction genes, some of which are derived from ERV insertions.
